# Supplementary figures and images for: A single microRNA miR-195 rescues the arrested B cell development induced by EBF1 deficiency
Source: eLife. 2026 Feb 6;13:RP101510. doi: 10.7554/eLife.101510 (PMC12880805; doi:10.7554/eLife.101510)

Fig. 4 FSD

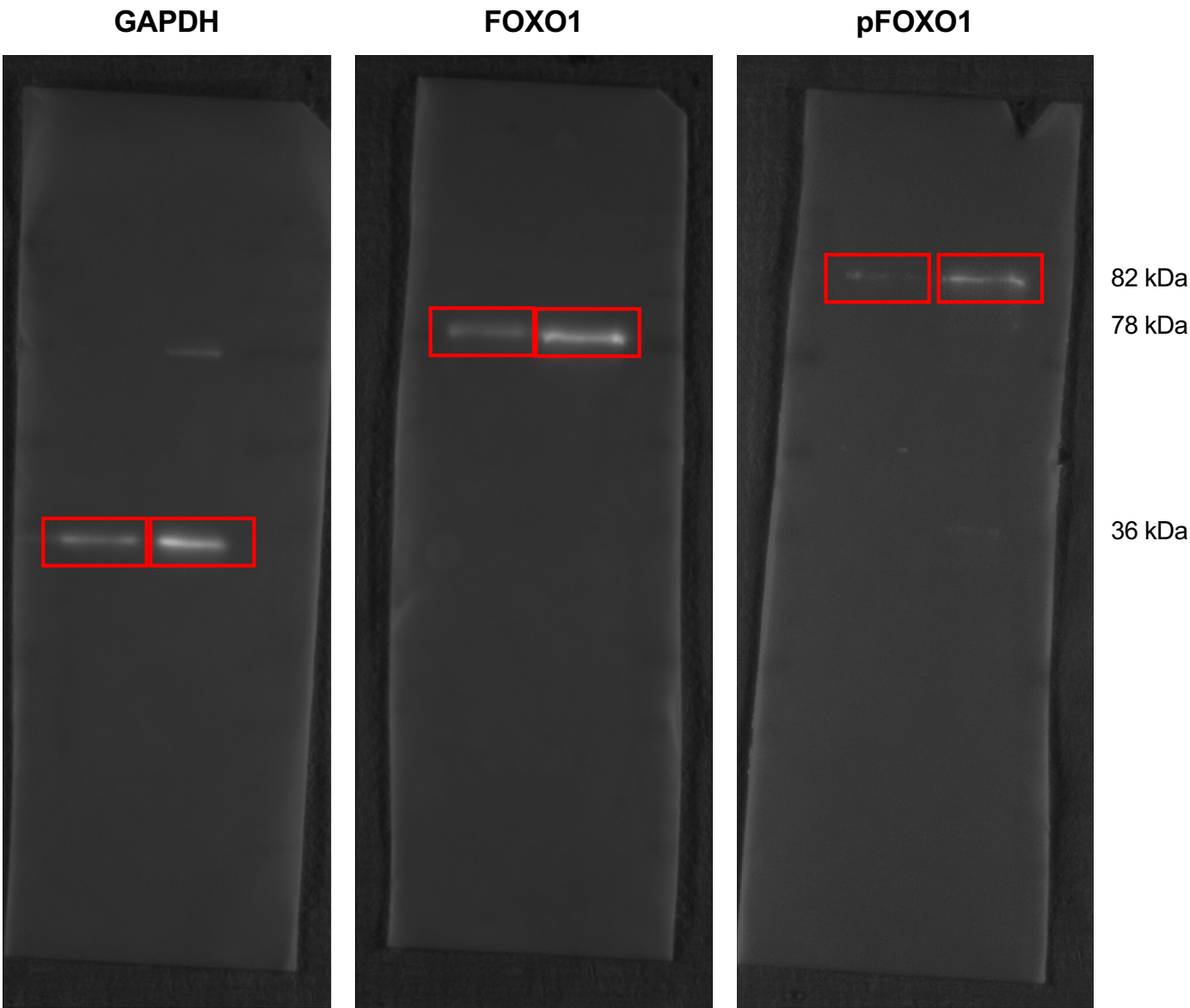

Supplement: Figure 4—source data 1. [file elife-101510-fig4-data1.zip › Figure4-source data 1/Figure4-source data 1.pdf]

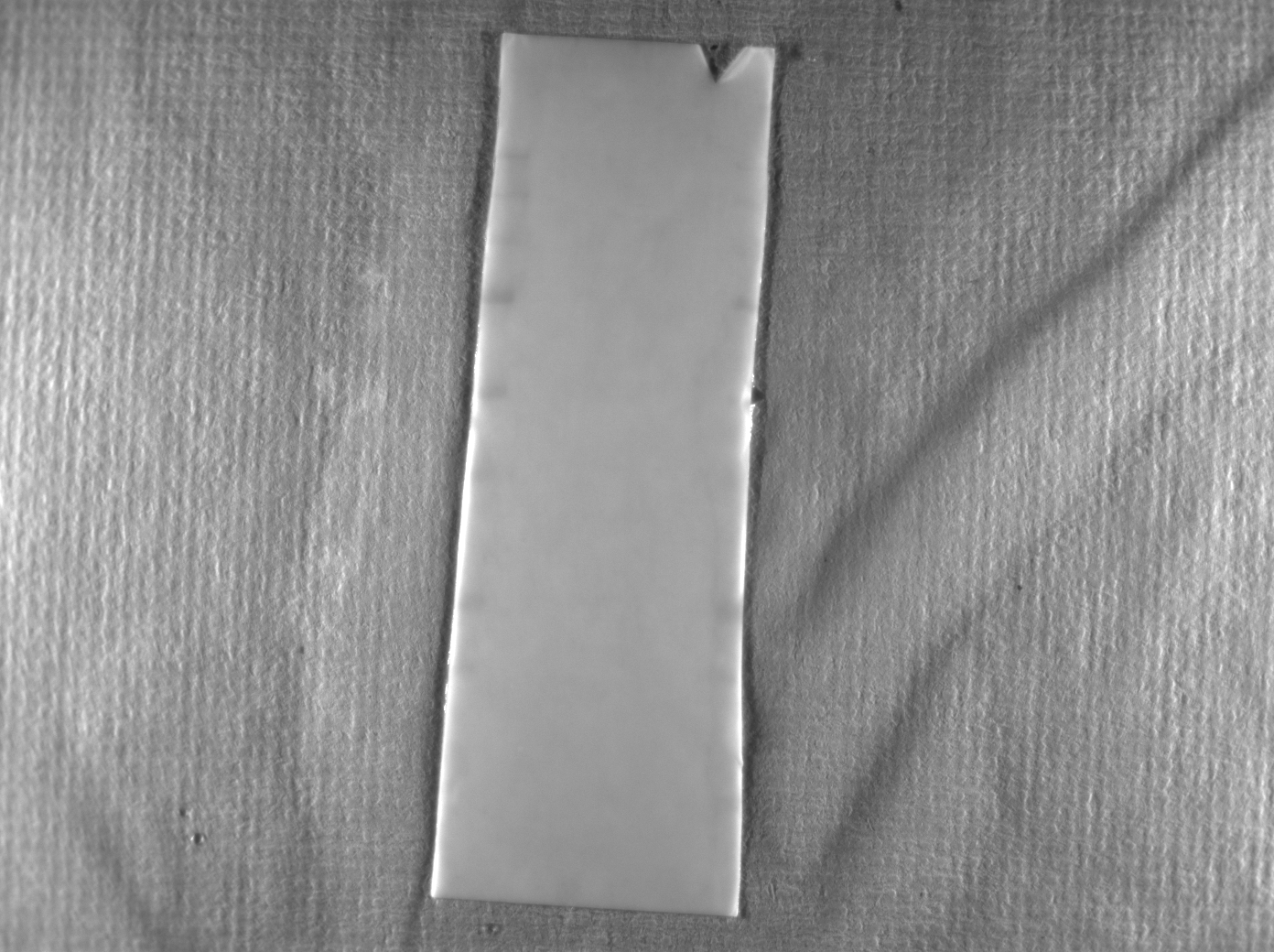

Supplement: Figure 4—source data 2. [file elife-101510-fig4-data2.zip › Figure4-source data 2/pFOXO1_memb.tif]

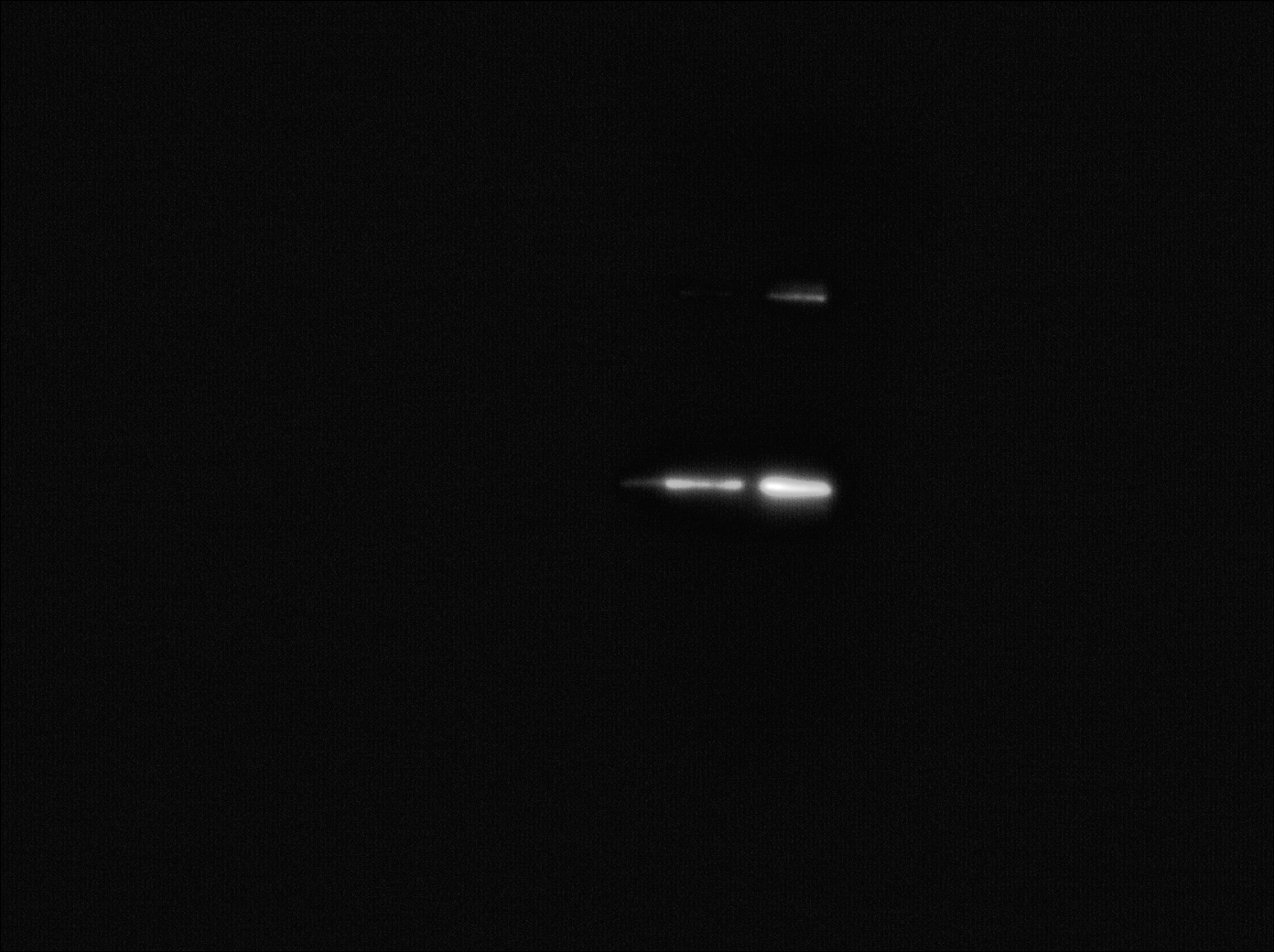

Supplement: Figure 4—source data 2. [file elife-101510-fig4-data2.zip › Figure4-source data 2/GAPDH_chemilumi.tif]

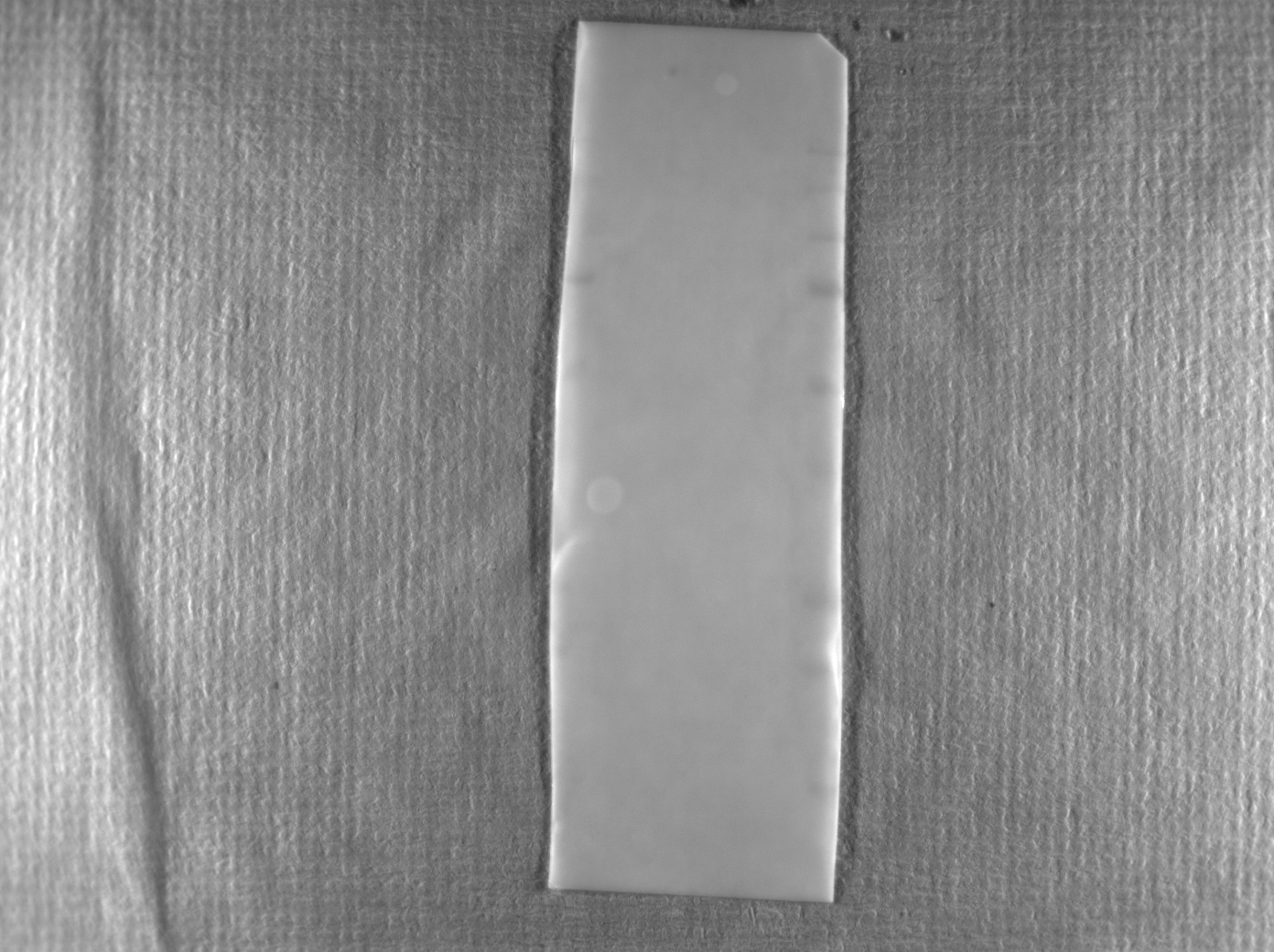

Supplement: Figure 4—source data 2. [file elife-101510-fig4-data2.zip › Figure4-source data 2/FOXO1_memb.tif]

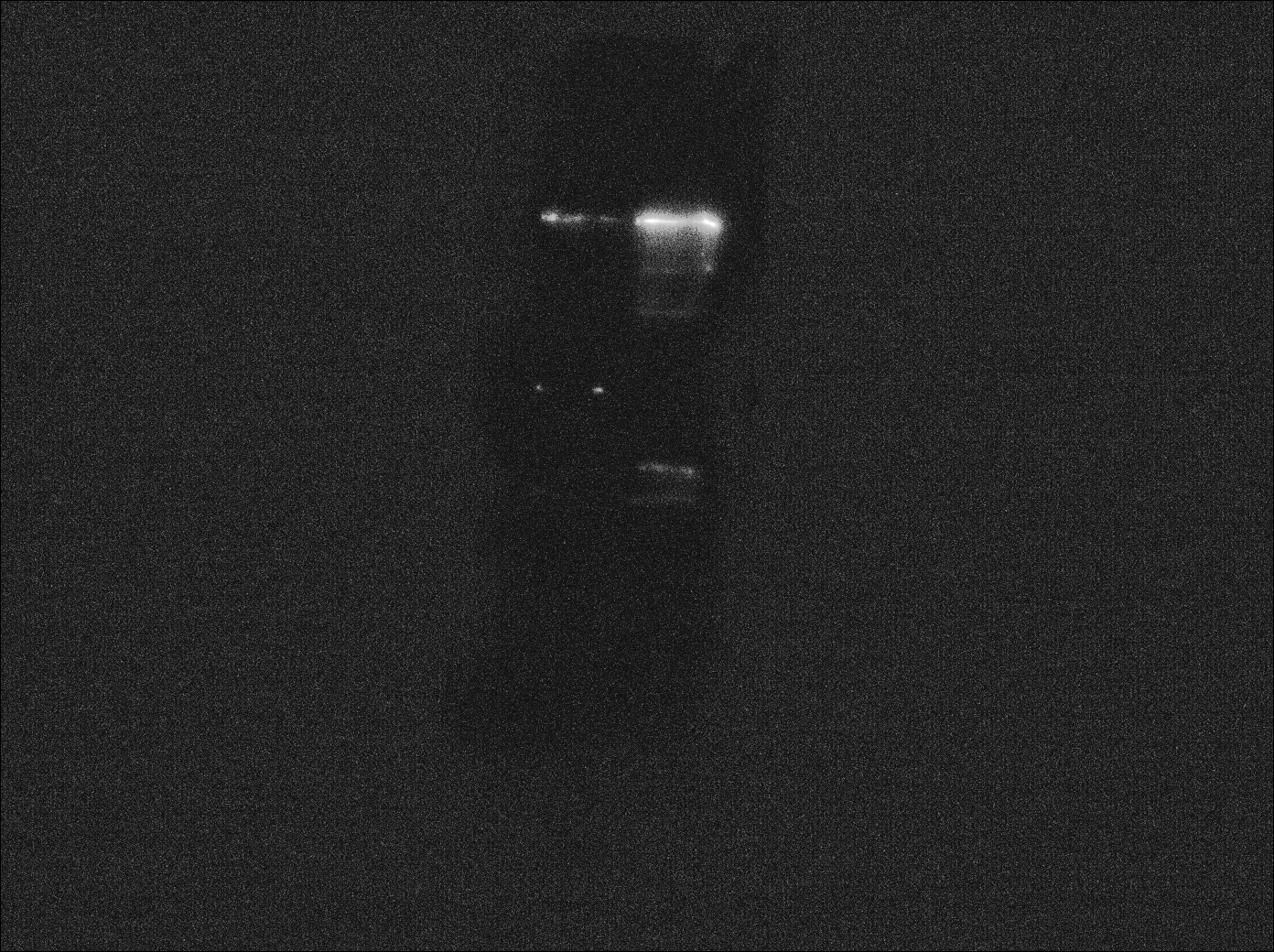

Supplement: Figure 4—source data 2. [file elife-101510-fig4-data2.zip › Figure4-source data 2/pFOXO1_chemilumi.tif]

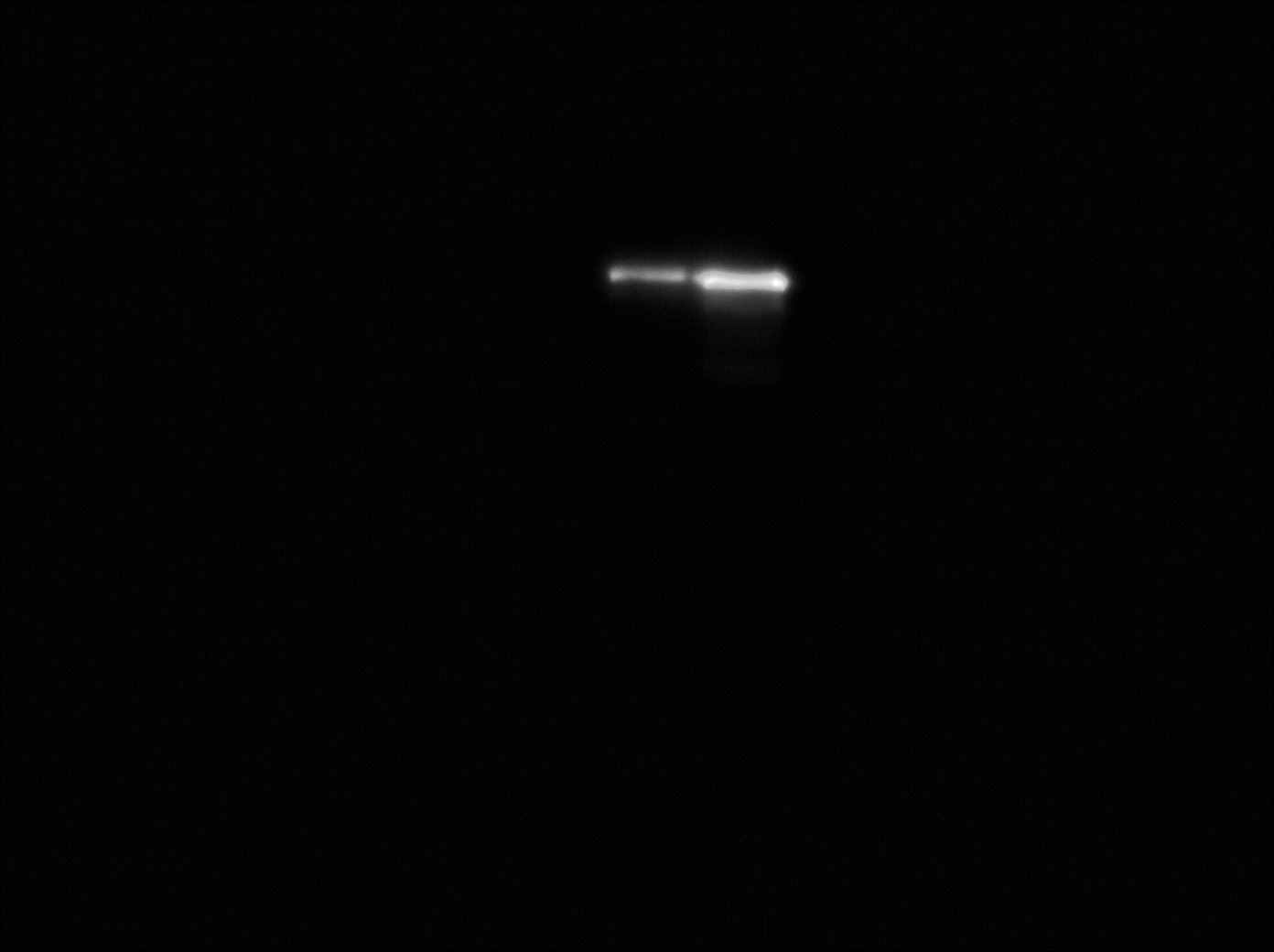

Supplement: Figure 4—source data 2. [file elife-101510-fig4-data2.zip › Figure4-source data 2/FOXO1_chemilumi.tif]

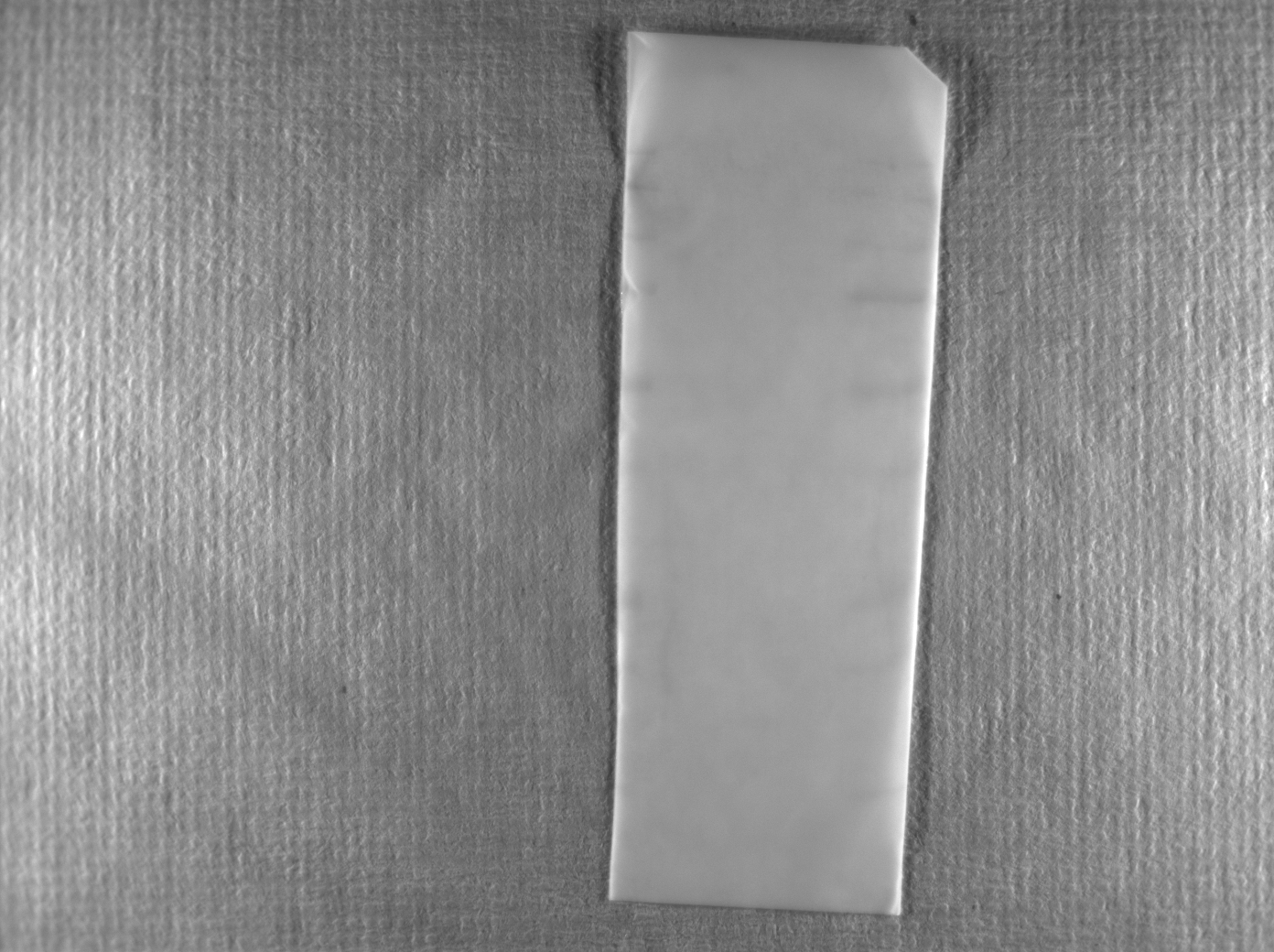

Supplement: Figure 4—source data 2. [file elife-101510-fig4-data2.zip › Figure4-source data 2/GAPDH_memb.tif]

Fig. 4-Fig. S1 FSD

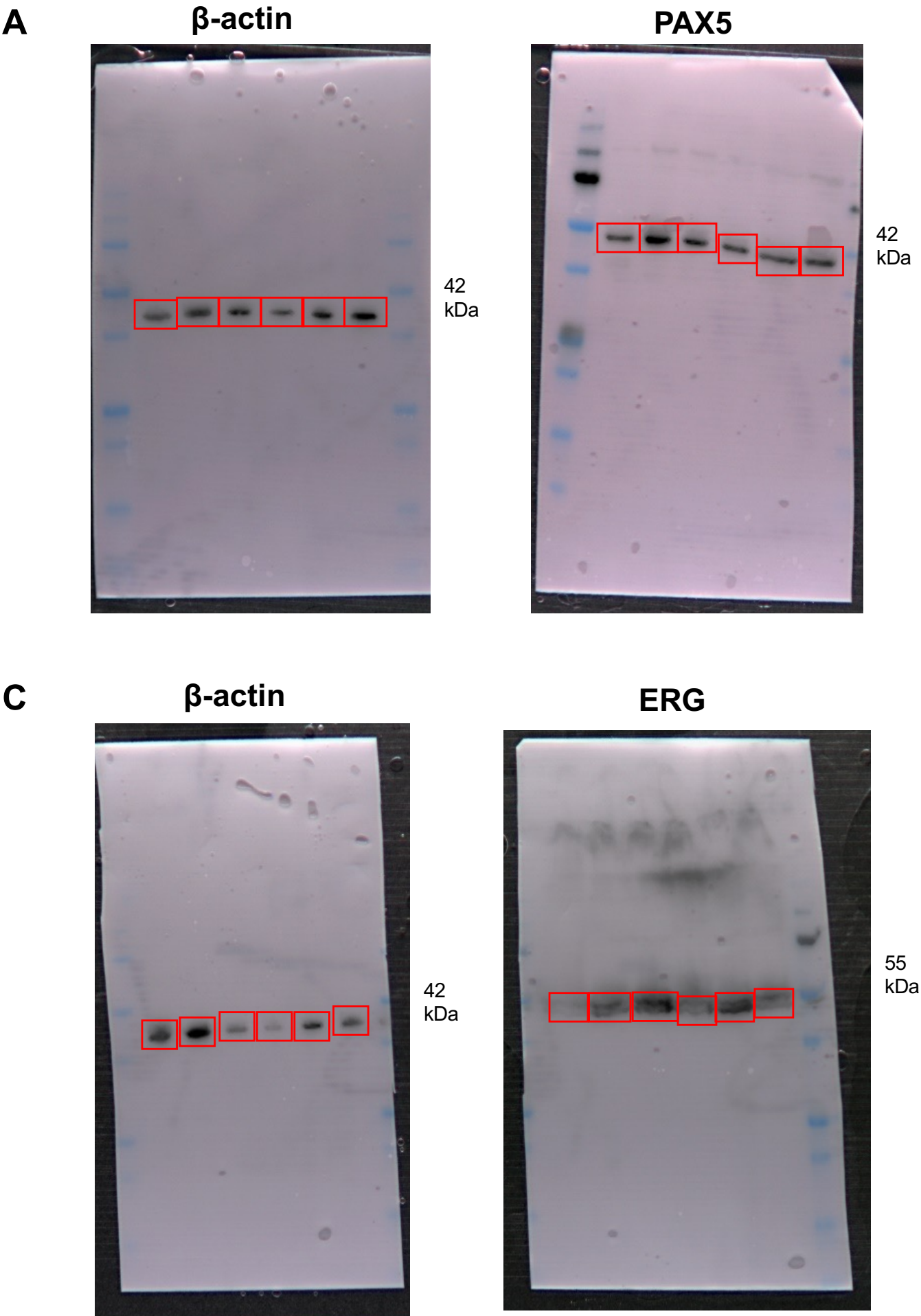

Supplement: Figure 4—figure supplement 1—source data 1. [file elife-101510-fig4-figsupp1-data1.zip › Figure4-figure supplement 1-source data 1/Figure4-FigureSupplement1-source data 1.pdf]

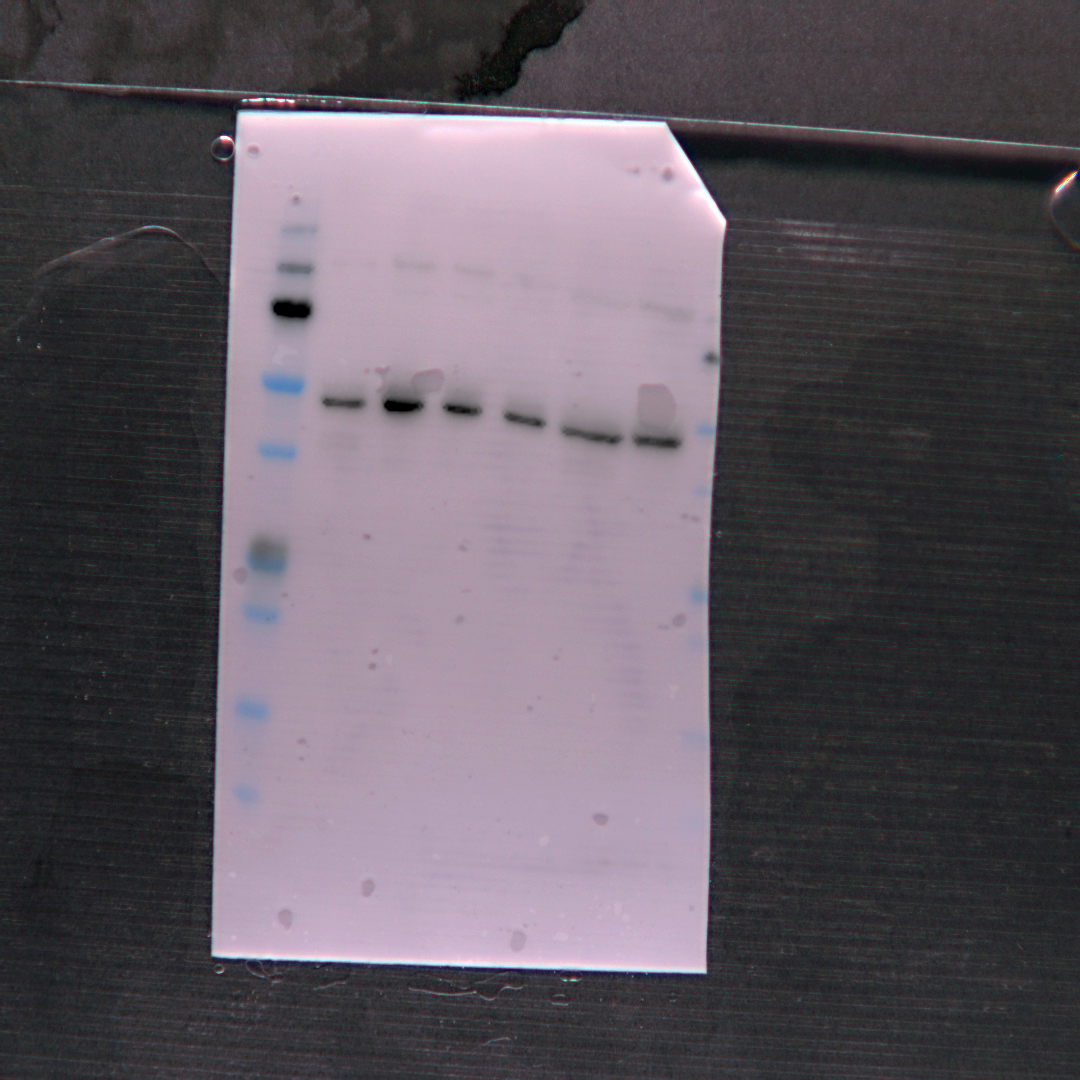

Supplement: Figure 4—figure supplement 1—source data 2. [file elife-101510-fig4-figsupp1-data2.zip › Figure4-figure supplement 1-source data 2/PAX5_merge.Tif]

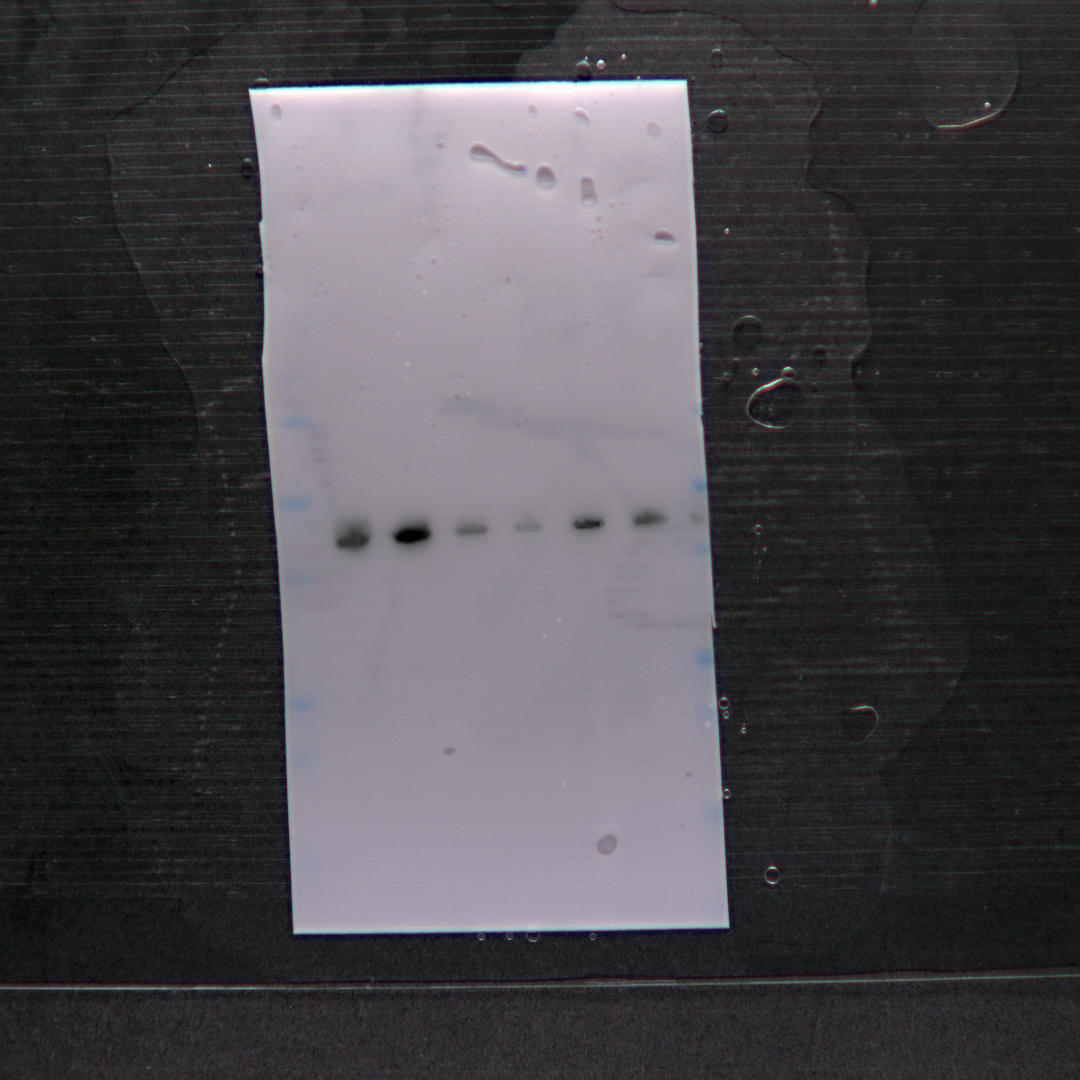

Supplement: Figure 4—figure supplement 1—source data 2. [file elife-101510-fig4-figsupp1-data2.zip › Figure4-figure supplement 1-source data 2/b-actin for ERG_merge.Tif]

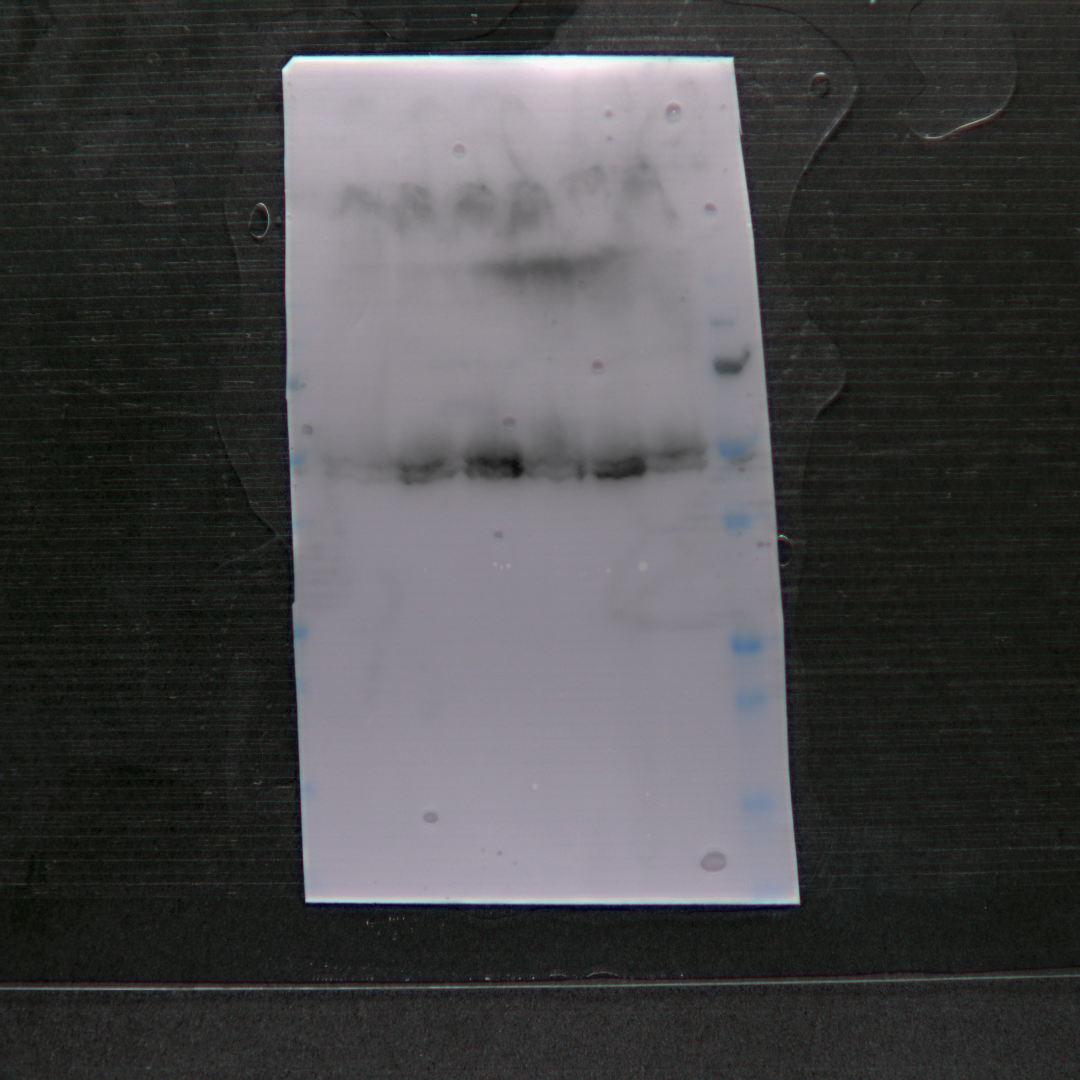

Supplement: Figure 4—figure supplement 1—source data 2. [file elife-101510-fig4-figsupp1-data2.zip › Figure4-figure supplement 1-source data 2/ERG_merge.Tif]

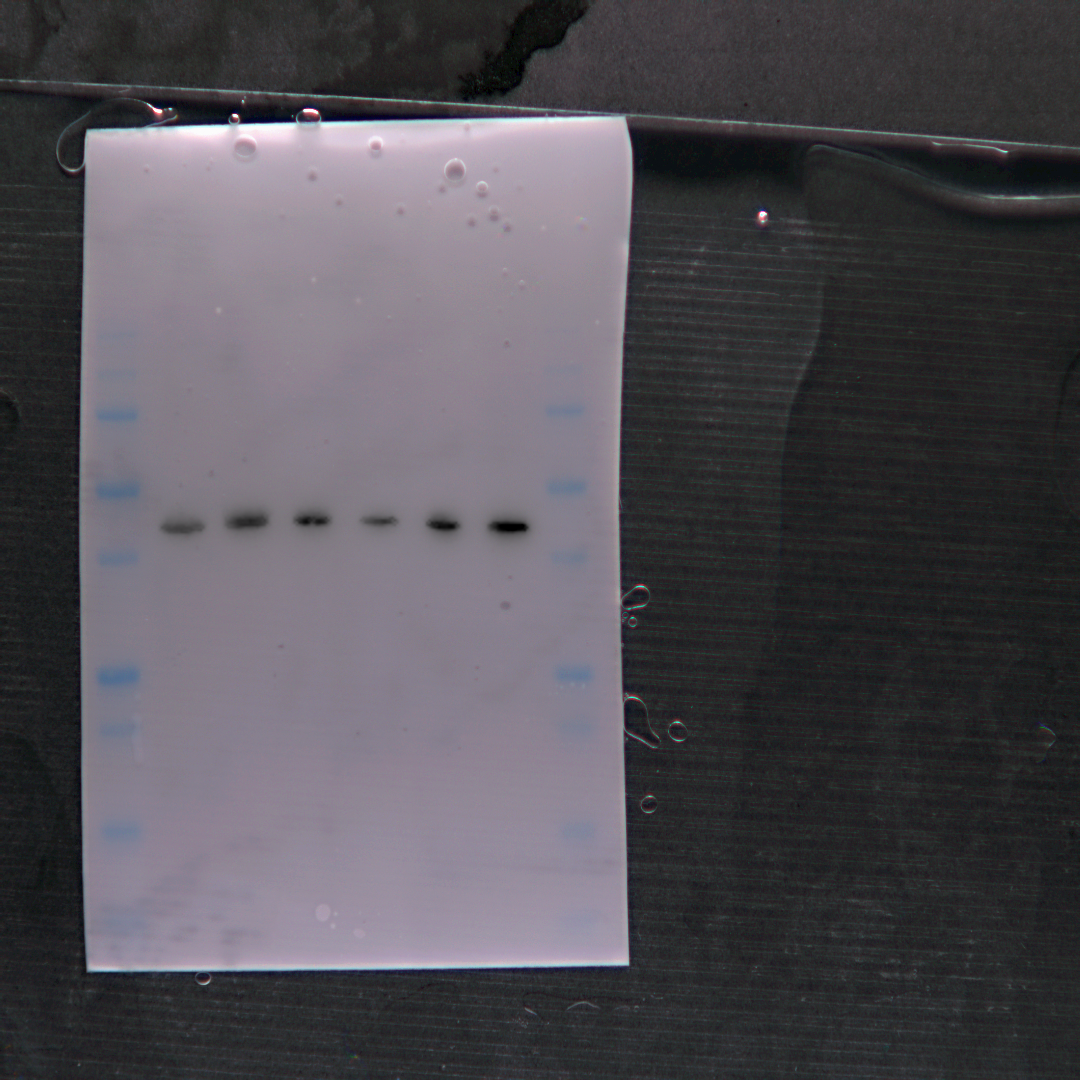

Supplement: Figure 4—figure supplement 1—source data 2. [file elife-101510-fig4-figsupp1-data2.zip › Figure4-figure supplement 1-source data 2/b-actin for PAX5_merge.Tif]

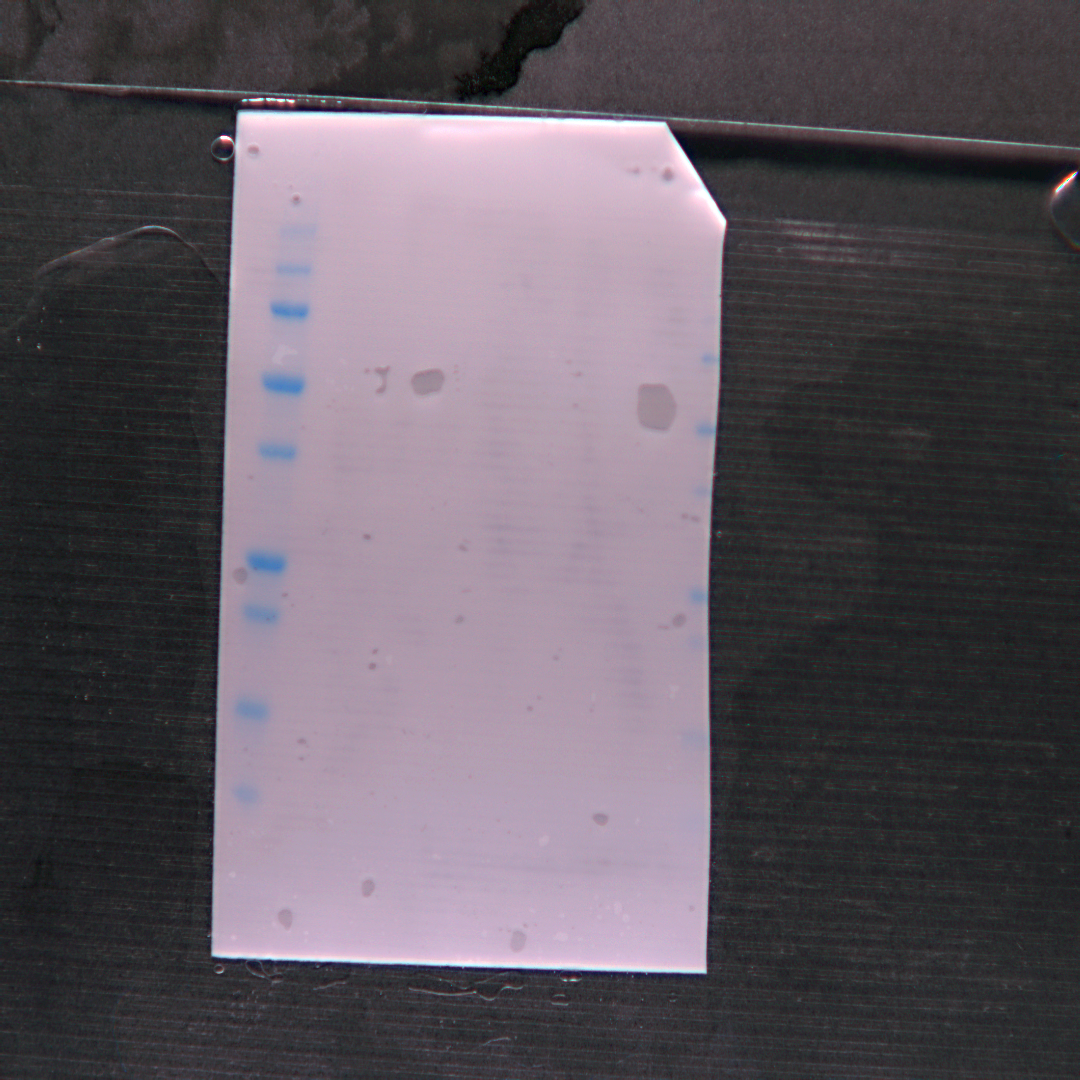

Supplement: Figure 4—figure supplement 1—source data 2. [file elife-101510-fig4-figsupp1-data2.zip › Figure4-figure supplement 1-source data 2/PAX5_memb.Tif]

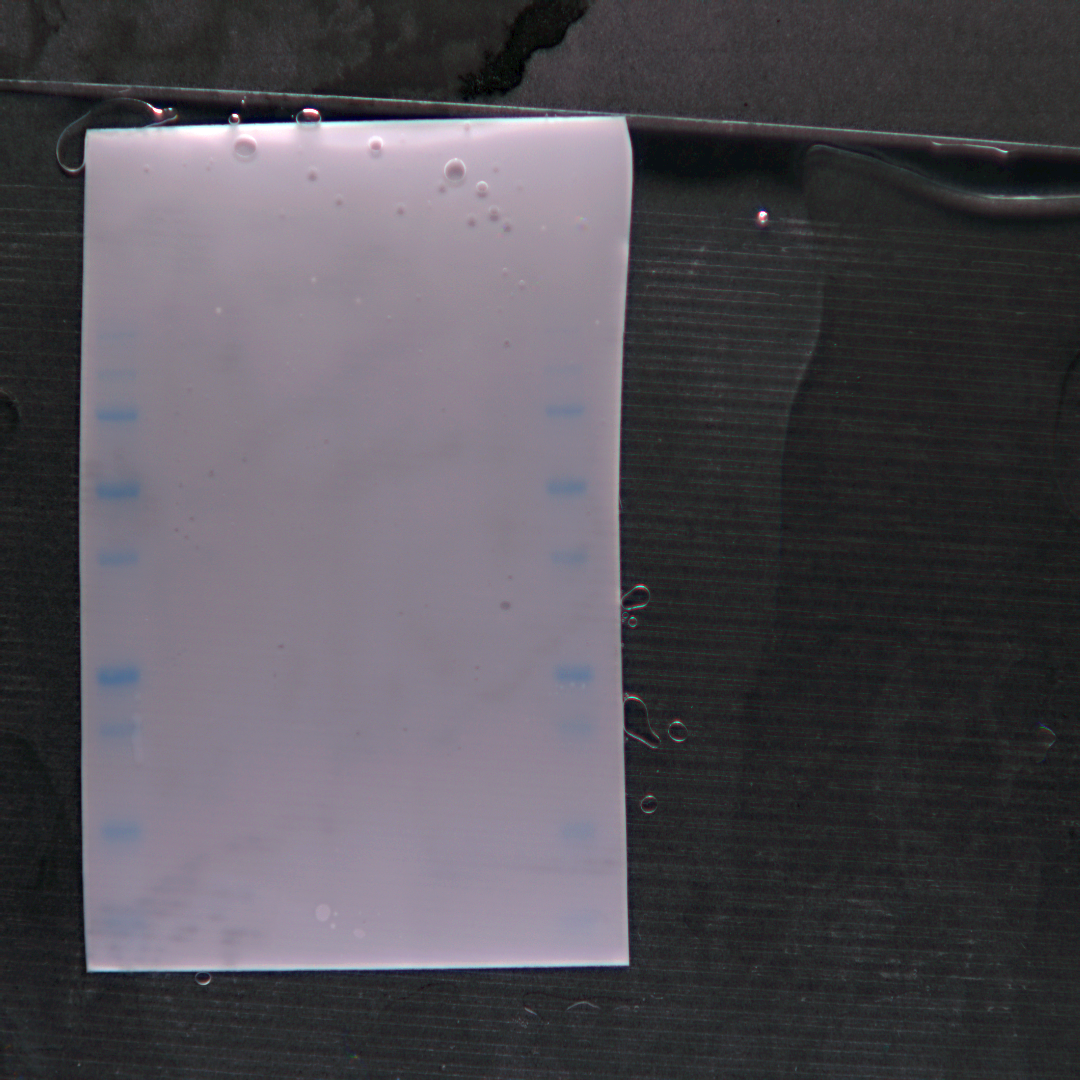

Supplement: Figure 4—figure supplement 1—source data 2. [file elife-101510-fig4-figsupp1-data2.zip › Figure4-figure supplement 1-source data 2/b-actin for PAX5_memb.Tif]

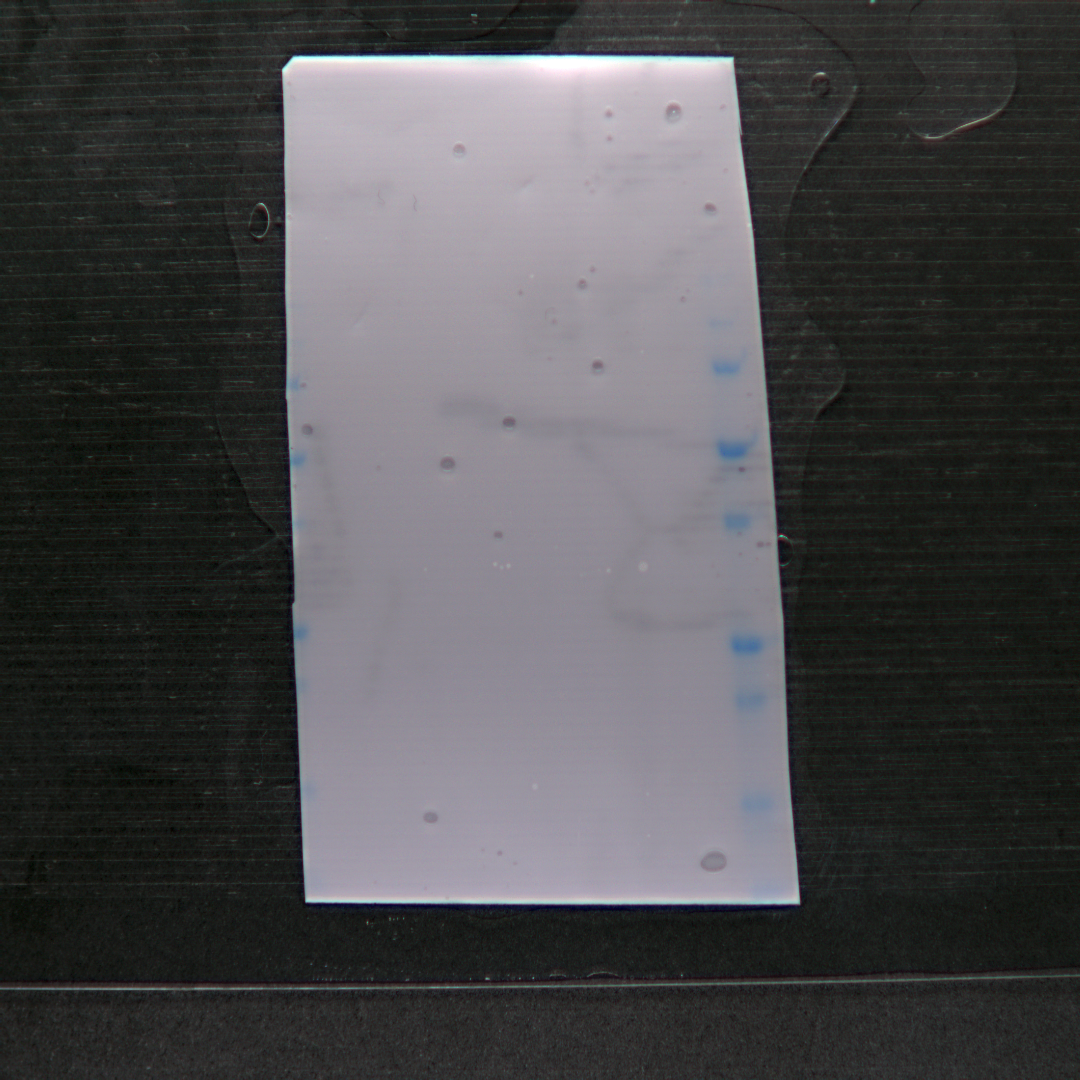

Supplement: Figure 4—figure supplement 1—source data 2. [file elife-101510-fig4-figsupp1-data2.zip › Figure4-figure supplement 1-source data 2/ERG_memb.Tif]

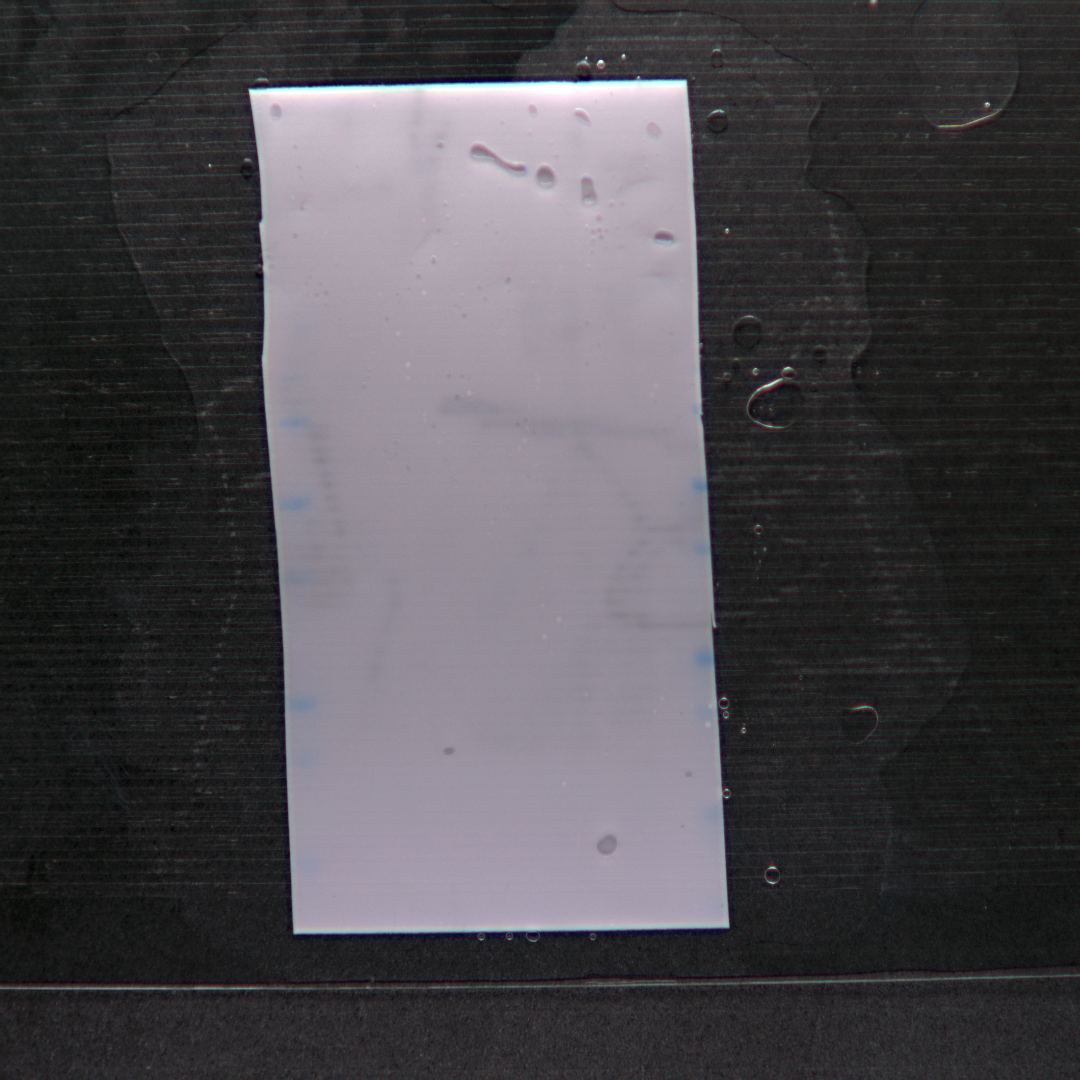

Supplement: Figure 4—figure supplement 1—source data 2. [file elife-101510-fig4-figsupp1-data2.zip › Figure4-figure supplement 1-source data 2/b-actin for ERG_memb.Tif]
