# Supplementary material for: A single microRNA miR-195 rescues the arrested B cell development induced by EBF1 deficiency
Source: eLife. 2026 Feb 6;13:RP101510. doi: 10.7554/eLife.101510 (PMC12880805; doi:10.7554/eLife.101510)
Supplement: Supplementary file 3 [file elife-101510-supp1.zip › Supplementary file 1.odt]

Supplementary File 1. Lists of high-p-value pathways detected by means of targetome analysis of human Mir195 with target scan and starBase in the KEGG pathway. 

| Term | Count | P-Value | Benjamini |
| --- | --- | --- | --- |
| Neurotrophin signaling pathway | 38 | 2.20E-08 | 6.20E-06 |
| MAPK signaling pathway | 62 | 4.20E-08 | 5.80E-06 |
| Insulin signaling pathway | 40 | 1.30E-07 | 1.20E-05 |
| mTOR signaling pathway | 23 | 3.10E-07 | 2.20E-05 |
| Renal cell carcinoma | 24 | 7.20E-07 | 4.00E-05 |
| Acute myeloid leukemia | 21 | 3.20E-06 | 1.50E-04 |
| RNA transport | 42 | 8.00E-06 | 3.20E-04 |
| AMPK signaling pathway | 33 | 1.00E-05 | 3.50E-04 |
| Proteoglycans in cancer | 46 | 1.50E-05 | 4.50E-04 |
| HTLV-I infection | 55 | 1.50E-05 | 4.30E-04 |
| Pathways in cancer | 76 | 1.60E-05 | 4.10E-04 |
| PI3K-Akt signaling pathway | 68 | 2.60E-05 | 6.00E-04 |
| Signaling pathways regulating pluripotency of stem cells | 35 | 3.10E-05 | 6.70E-04 |
| Prostate cancer | 25 | 6.50E-05 | 1.30E-03 |
| Endocytosis | 53 | 8.00E-05 | 1.50E-03 |
| Insulin resistance | 28 | 1.20E-04 | 2.10E-03 |
| Focal adhesion | 44 | 1.50E-04 | 2.40E-03 |
| TNF signaling pathway | 27 | 2.20E-04 | 3.40E-03 |
| Regulation of actin cytoskeleton | 44 | 2.50E-04 | 3.70E-03 |
| Oocyte meiosis | 27 | 3.60E-04 | 4.90E-03 |
| Phosphatidylinositol signaling system | 25 | 3.90E-04 | 5.20E-03 |
| Progesterone-mediated oocyte maturation | 23 | 4.20E-04 | 5.30E-03 |
| Pancreatic cancer | 19 | 4.20E-04 | 5.10E-03 |
| Lysosome | 28 | 8.50E-04 | 9.80E-03 |
| FoxO signaling pathway | 30 | 9.30E-04 | 1.00E-02 |
| HIF-1 signaling pathway | 24 | 9.70E-04 | 1.00E-02 |
| Adherens junction | 19 | 1.30E-03 | 1.40E-02 |
| Ubiquitin mediated proteolysis | 30 | 1.30E-03 | 1.30E-02 |
| Wnt signaling pathway | 30 | 1.50E-03 | 1.40E-02 |
| Hippo signaling pathway | 32 | 1.60E-03 | 1.50E-02 |
| Epstein-Barr virus infection | 38 | 1.60E-03 | 1.40E-02 |
| Non-small cell lung cancer | 16 | 1.80E-03 | 1.60E-02 |
| mRNA surveillance pathway | 22 | 2.00E-03 | 1.70E-02 |
| Ras signaling pathway | 43 | 2.10E-03 | 1.70E-02 |
| Cell cycle | 27 | 2.70E-03 | 2.10E-02 |
| Shigellosis | 17 | 2.80E-03 | 2.20E-02 |
| Hepatitis B | 30 | 3.30E-03 | 2.50E-02 |
| Choline metabolism in cancer | 23 | 3.40E-03 | 2.50E-02 |
| Prolactin signaling pathway | 18 | 3.40E-03 | 2.40E-02 |
| Chronic myeloid leukemia | 18 | 4.00E-03 | 2.80E-02 |
| Small cell lung cancer | 20 | 4.60E-03 | 3.10E-02 |
