# Supplementary material for: A single microRNA miR-195 rescues the arrested B cell development induced by EBF1 deficiency
Source: eLife. 2026 Feb 6;13:RP101510. doi: 10.7554/eLife.101510 (PMC12880805; doi:10.7554/eLife.101510)
Supplement: Supplementary file 4 [file elife-101510-supp2.zip › Supplementary file 2.odt]

Supplementary File 2. Lists of high-p-value pathways detected by means of targetome analysis of murine Mir195 with target scan and starBase in the KEGG pathway.

| Term | Count | P-Value | Benjamini |
| --- | --- | --- | --- |
| Insulin signaling pathway | 18 | 5.60E-06 | 1.40E-03 |
| Protein processing in endoplasmic reticulum | 19 | 1.70E-05 | 2.10E-03 |
| Focal adhesion | 21 | 2.80E-05 | 2.30E-03 |
| Pancreatic cancer | 11 | 6.70E-05 | 4.10E-03 |
| PI3K-Akt signaling pathway | 28 | 6.90E-05 | 3.40E-03 |
| Neurotrophin signaling pathway | 15 | 7.20E-05 | 2.90E-03 |
| Cell cycle | 15 | 8.60E-05 | 3.00E-03 |
| Toxoplasmosis | 14 | 1.30E-04 | 3.90E-03 |
| TGF-beta signaling pathway | 12 | 1.50E-04 | 4.00E-03 |
| Prostate cancer | 12 | 2.00E-04 | 4.90E-03 |
| Signaling pathways regulating pluripotency of stem cells | 15 | 2.70E-04 | 6.00E-03 |
| Colorectal cancer | 10 | 3.10E-04 | 6.30E-03 |
| Renal cell carcinoma | 10 | 4.40E-04 | 8.30E-03 |
| Phosphatidylinositol signaling system | 12 | 4.70E-04 | 8.20E-03 |
| Pathways in cancer | 28 | 5.30E-04 | 8.50E-03 |
| HTLV-I infection | 22 | 5.80E-04 | 8.80E-03 |
| Small cell lung cancer | 11 | 5.80E-04 | 8.30E-03 |
| FoxO signaling pathway | 14 | 6.90E-04 | 9.30E-03 |
| Progesterone-mediated oocyte maturation | 11 | 7.70E-04 | 9.80E-03 |
| Prolactin signaling pathway | 10 | 8.40E-04 | 1.00E-02 |
| Hepatitis B | 14 | 1.50E-03 | 1.80E-02 |
| Endometrial cancer | 8 | 1.90E-03 | 2.10E-02 |
| Hippo signaling pathway | 14 | 2.10E-03 | 2.20E-02 |
| Measles | 13 | 2.50E-03 | 2.50E-02 |
| Hepatitis C | 13 | 2.50E-03 | 2.50E-02 |
| Non-small cell lung cancer | 8 | 2.90E-03 | 2.80E-02 |
| HIF-1 signaling pathway | 11 | 3.00E-03 | 2.80E-02 |
| Chronic myeloid leukemia | 9 | 3.20E-03 | 2.80E-02 |
| Regulation of lipolysis in adipocytes | 8 | 3.20E-03 | 2.80E-02 |
| Ubiquitin mediated proteolysis | 13 | 3.50E-03 | 2.90E-02 |
| Sphingolipid signaling pathway | 12 | 3.60E-03 | 2.90E-02 |
| AMPK signaling pathway | 12 | 4.30E-03 | 3.30E-02 |
| Epstein-Barr virus infection | 16 | 7.30E-03 | 5.40E-02 |
| Acute myeloid leukemia | 7 | 1.20E-02 | 8.60E-02 |
| Oocyte meiosis | 10 | 1.30E-02 | 9.20E-02 |
| Endocytosis | 18 | 1.50E-02 | 1.00E-01 |
| Rap1 signaling pathway | 15 | 1.60E-02 | 1.00E-01 |
