# Supplementary material for: A single microRNA miR-195 rescues the arrested B cell development induced by EBF1 deficiency
Source: eLife. 2026 Feb 6;13:RP101510. doi: 10.7554/eLife.101510 (PMC12880805; doi:10.7554/eLife.101510)
Supplement: Supplementary file 5 [file elife-101510-supp3.zip › Supplementary file 3.odt]

Supplementary File 3. List of genes with detected peaks in the DNA region common in the three analyses.

| Gene names having open chromatin peak(s) in  WT CD19+ vs Ebf1 KO CD19- and WT CD19+(~IgM+) vs WT CD19- and Mir195 CD19+ vs EMPTY CD19- |
| --- |
| Ablim1, Acoxl, Alk, Ankrd6, Atg7, B230118H07Rik, BC003331, Bfsp1, Blnk, Cacna1d, Capn11, Cdc42se2, Ciita, Cmip, Dennd4a, Dio2, Ebf1, Eif2ak3, Entpd5, Epb41l2, Erg, Foxn3, Foxp1, Gabrr2, Gpr20, Grik4, Hivep3, Irf8, Itga9, Itpa, Itpr2, Jakmip1, Kalrn, Ldlrad4, Lipc, Med13l, Mgll, Mical3, Myo1d, Nav2, Nfia, Nsmce2, Osbpl10, Pakap, Pax5, Pde2a, Pde4c, Pgm5, Plxnc1, Pold1, Prlr, Rarres1, Rassf5, Rd3, Rnf41, Runx1, Ryr2, Scn4a, Sh3bp5, Slc39a11, Spink2, Stxbp1, Tbc1d1, Tg, Thada, Tmem64, Trappc9, Trio, Ubac2, Ube2h, Vps8, Whrn, 2410004B18Rik |
